# Very early versus early exercise rehabilitation after intracerebral hemorrhage: A systematic review and meta-analysis

**DOI:** 10.1097/MD.0000000000050253

**Published:** 2026-08-21

**Authors:** Taiqiang Kan, Lining Ding, Siqi Wang, Tong Li, Yunfei Li, Zhongqin He, Chun Shi, Chenbang Ma, Zhuang Li, Bensi Zhang

**Affiliations:** a College of Basic Medicine, Dali University, Dali, Yunnan, China; b Xiajin County People’s Hospital, Dezhou, Shandong, China; c College of Clinical Medicine, Dali University, Dali, Yunnan, China; d College of Dental Medicine, Western University of Health Sciences, Pomona, CA.

**Keywords:** early mobilization, exercise rehabilitation, ICH, meta-analysis, stroke rehabilitation, systematic review

## Abstract

**Background:**

The optimal timing of rehabilitation initiation after intracerebral hemorrhage (ICH) remains uncertain. This systematic review and meta-analysis compared the efficacy and safety of very early versus early exercise rehabilitation after ICH.

**Methods:**

Chinese Biomedical Literature Database, China National Knowledge Infrastructure, Chinese Science and Technology Journal Database/VIP Database, Wanfang, PubMed, Embase, the Cochrane Library, Web of Science, ClinicalTrials.gov, and the Chinese Clinical Trial Registry were searched from inception to October 10, 2023. Randomized controlled trials comparing very early with early exercise rehabilitation after ICH were included. Two reviewers independently screened studies, extracted data, and assessed risk of bias. Continuous outcomes were pooled as mean differences (MDs) with 95% confidence intervals (CIs). Certainty of evidence was assessed using the Grading of Recommendations Assessment, Development and Evaluation approach. Primary outcomes included the National Institutes of Health Stroke Scale, Fugl-Meyer Assessment, Modified Barthel Index, and Barthel Index.

**Results:**

Seventeen randomized controlled trials involving 1396 participants were included. Compared with early rehabilitation, very early rehabilitation was associated with better outcomes for National Institutes of Health Stroke Scale (MD = −3.89, 95% CI: −5.00 to −2.78), Fugl-Meyer Assessment (MD = 10.24, 95% CI: 6.80–13.68), Modified Barthel Index (MD = 0.92, 95% CI: 0.73–1.11), and Barthel Index (MD = 1.89, 95% CI: 1.70–2.08). Substantial heterogeneity was observed across the pooled analyses. Standardized safety outcomes, including rebleeding, hemodynamic instability, and falls, were insufficiently reported for quantitative synthesis. The certainty of evidence was low for all outcomes.

**Conclusion:**

In clinically stable patients with ICH, very early exercise rehabilitation may be associated with better neurological and functional recovery than rehabilitation initiated later within the early phase. However, the findings should be interpreted cautiously because of substantial heterogeneity, incomplete safety reporting, and low certainty of evidence.

## 1. Introduction

Intracerebral hemorrhage (ICH) is a severe subtype of stroke characterized by acute onset, rapid progression, and high mortality and disability. Although ICH accounts for only a minority of all stroke cases, it is associated with disproportionately poor outcomes, including high early mortality and substantial long-term functional impairment.^[1–3]^

In addition to medical and surgical treatment, rehabilitation is a key component of post-ICH care. Exercise-based rehabilitation may improve neurological recovery, reduce disability, and enhance activities of daily living by promoting motor relearning and preventing complications associated with immobility.^[4–7]^

Experimental and clinical studies suggest that early rehabilitation may take advantage of a critical period of neuroplasticity after brain injury, during which cortical reorganization and functional recovery may be more responsive to intervention.^[6,8–10]^ However, prolonged bed rest has traditionally been common in clinical practice, especially in patients perceived to be medically fragile, despite its association with deconditioning, muscle atrophy, pressure injury, pulmonary complications, and delayed recovery.^[11,12]^

The timing of rehabilitation initiation after ICH remains controversial. On the 1 hand, very early mobilization may facilitate neurological recovery by exploiting the early recovery window and reducing the adverse effects of immobility.^[7,12–14]^ On the other hand, initiating rehabilitation too early, particularly in the hyperacute phase, may raise concerns regarding blood pressure instability, impaired cerebral autoregulation, hematoma expansion, rebleeding, falls, and other adverse events.^[4,7,13]^ As a result, clinicians often remain cautious when determining when to begin structured exercise rehabilitation after ICH.

Although early rehabilitation is widely recommended in stroke care, there is still no clear consensus regarding the optimal initiation window after ICH.^[4,7,13]^ In particular, the comparative efficacy and safety of very early versus early exercise rehabilitation remain insufficiently clarified. Therefore, this systematic review and meta-analysis was conducted to compare the efficacy and safety of very early versus early exercise rehabilitation after ICH and to provide evidence to inform rehabilitation timing in clinical practice.

## 2. Methods

### 2.1. Search strategy

A systematic literature search was conducted in the Chinese Biomedical Literature Database, China National Knowledge Infrastructure, Chinese Science and Technology Journal Database/VIP Database, Wanfang Database, PubMed, Embase, the Cochrane Library, Web of Science, ClinicalTrials.gov, and the Chinese Clinical Trial Registry. The search period extended from database inception to October 10, 2023. A combination of subject headings and free-text terms was used, including terms related to ICH, rehabilitation, mobilization, exercise training, and timing of intervention. The reference lists of relevant articles were also manually screened to identify additional eligible studies.

In response to the editorial request, we performed a post-search evidence check after receiving the revision comments. Using the original search strategy, we screened for randomized trials published after the prespecified search date of October 10, 2023. This check identified 3 potentially relevant post-search randomized trials. These trials reported findings directionally consistent with the pooled estimates of the present meta-analysis; that is, earlier rehabilitation initiation was associated with better neurological or functional outcomes, including National Institutes of Health Stroke Scale (NIHSS), Fugl-Meyer Assessment (FMA), Barthel Index (BI), or related functional measures. These trials were treated as post-search evidence and were considered descriptively when interpreting the findings.

However, these post-search trials differed from the original prespecified contrast in their timing comparators, including 48 versus 72 hours, 48 hours versus 7 days, and 12 versus 48 hours. In addition, 1 trial used a non-NIHSS neurological deficit scale and reported unusually narrow dispersion values for several functional outcomes, raising concerns about compatibility with the present pooled outcomes and statistical weighting. Because formal integration of these studies would require a full update of the systematic review process, including renewed dual-reviewer study selection, data extraction, risk-of-bias assessment, Grading of Recommendations Assessment, Development and Evaluation (GRADE) evaluation, and reanalysis of pooled estimates, these post-search trials were not entered into the quantitative synthesis and were considered descriptively. The primary pooled estimates, risk-of-bias assessment, and GRADE ratings therefore reflect the evidence available within the original prespecified search period.

### 2.2. Eligibility criteria

Studies were included if they met the following criteria:

1. randomized controlled trials;
2. adult patients with ICH confirmed by imaging or clearly defined diagnostic criteria in the original study;
3. comparison of very early exercise rehabilitation versus early exercise rehabilitation after ICH;
4. both groups received comparable basic medical or surgical care, with the main difference being the timing of rehabilitation initiation;
5. at least one of the following outcomes was reported: NIHSS, FMA, Modified Barthel Index (MBI), BI, or safety-related outcomes.

For the purposes of this review, very early rehabilitation was operationally defined as rehabilitation initiated within 1 week after admission or onset, whereas early rehabilitation was defined as rehabilitation initiated within 1 month. Because the included studies used varying initiation time points within these broad windows, these categories were considered study-level operational groupings rather than biologically uniform phases.

Studies were excluded if they were non-randomized studies, reviews, meta-analyses, conference abstracts without sufficient data, animal experiments, duplicate publications, or studies with incomplete outcome data that could not be extracted.

### 2.3. Study selection and data extraction

Two reviewers independently screened titles and abstracts, assessed full texts for eligibility, and extracted data using a predefined form. Disagreements were resolved through discussion, and a 3rd reviewer was consulted when necessary.

The following data were extracted: 1st author, publication year, sample size, participant characteristics, rehabilitation timing, intervention content, intervention duration, outcome measures, and information required for risk-of-bias assessment.

### 2.4. Risk-of-bias assessment

The methodological quality of the included studies was assessed independently by 2 reviewers using the Cochrane risk-of-bias tool.^[15]^ The following domains were evaluated: random sequence generation, allocation concealment, blinding, completeness of outcome data, selective reporting, and other potential sources of bias. Any disagreements were resolved through discussion or consultation with a 3rd reviewer.

### 2.5. Statistical analysis

Meta-analyses were performed using Review Manager 5.4 (The Cochrane Collaboration) and Stata 18 (StataCorp LLC, College Station, Texas). Continuous outcomes were pooled as mean differences (MDs) with 95% confidence intervals (CIs). Statistical heterogeneity was assessed using the *I*^2^ statistic and the corresponding *P* value. When substantial heterogeneity was present (*I*^2^ > 50%), a random-effects model was used; otherwise, a fixed-effects model was applied. Sensitivity analysis was conducted when appropriate to evaluate the stability of the pooled results. Safety outcomes were summarized descriptively because reported data were insufficient and too heterogeneous for quantitative pooling. Statistical significance was set at *P* < .05.

### 2.6. Certainty of evidence assessment

The certainty of evidence for each primary outcome was assessed using the GRADE approach. Evidence certainty was rated as high, moderate, low, or very low based on risk of bias, inconsistency, indirectness, imprecision, and publication bias. A summary-of-findings table was generated for NIHSS, FMA, MBI, and BI.

### 2.7. Ethical approval

Ethical approval and informed consent were not required for this study because this systematic review and meta-analysis was based exclusively on previously published aggregate data.

## 3. Results

### 3.1. Study selection

**Figure 1.**
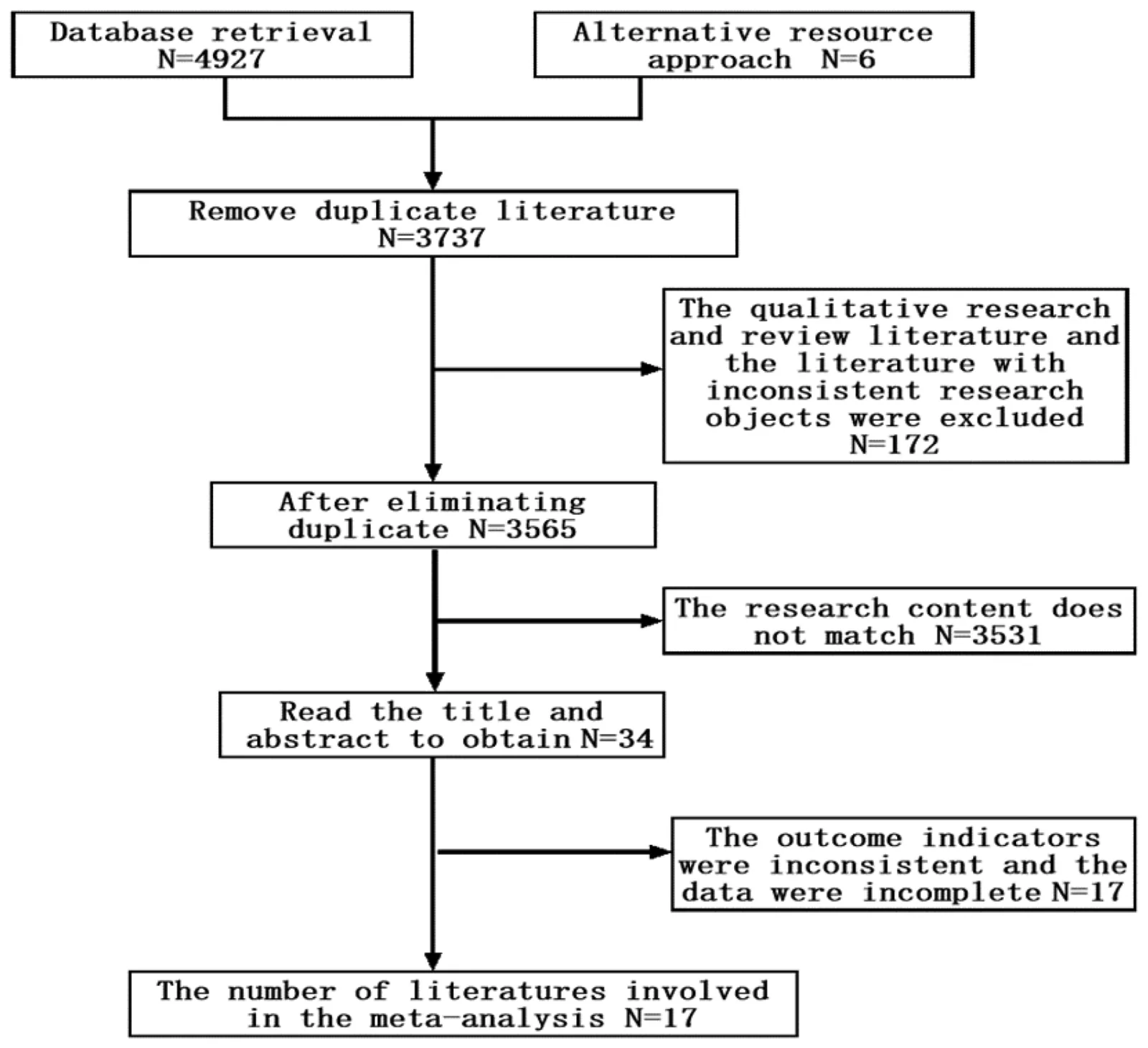
Literature screening process.

A total of 4933 records were identified, including 4927 records retrieved from databases and 6 records identified through alternative sources. After removal of duplicates, 3737 records remained. Of these, 172 qualitative studies, review articles, and studies with inconsistent research populations were excluded, leaving 3565 records for further screening. After title and abstract screening, 3531 records were excluded because their research content did not meet the eligibility criteria, leaving 34 articles for further assessment. Subsequently, 17 articles were excluded because the outcome measures were inconsistent with the predefined outcomes or the available data were incomplete. Ultimately, 17 studies were included in the meta-analysis. The study selection process is shown in Figure 1. .

### 3.2. Study characteristics

A total of 17 randomized controlled trials published between 2012 and 2023 were included.^[16–32]^ Across the included studies with available age data, the mean age of participants was approximately 64 years. The intervention groups were classified according to rehabilitation timing as a very early rehabilitation group (rehabilitation initiated within 1 week of admission or onset) and an early rehabilitation group (rehabilitation initiated within 1 month). In general, the rehabilitation programs included joint mobilization, upper-limb training, balance training, positioning, and activities related to self-care and daily living. The main reported outcomes included NIHSS, FMA, MBI, and BI. Detailed characteristics of the included studies are shown in Table 1.

**Table 1:** Basic characteristics of the included studies.

Author	Year	Control/experimental (sample)	Control (age)	Experimental (age)	Control intervention time	Experimental intervention time	Treatment time	Outcome index
Zhang WL	2018	33/33	63.6 ± 14.8	63.5 ± 14.6	72 h–2 wk	72 h	6 wk	①②
Huang QY	2016	44/22	68.15 ± 6.52	68.3 ± 5.6	72 h–4 wk	Within 72 h	6 wk	①②
Zhu Yan	2017	48/24	62.95 ± 3.57	62.3 ± 4.2	1–3 mo	Immediate	Unclear	②③④
Li L	2018	30/30	50.4 ± 4.3	51.4 ± 4.5	4 wk	Immediate	4 wk	①②④
Yang Q	2015	70/70	65 ± 5.5	64 ± 3.5	3 wk	72 h	8 wk	①②④
Yin YP	2015	41/41	65.4 ± 2.5	68.2 ± 3.1	1–3 wk	48 h	6 wk	②④
Liu AJ	2018	52/52	59.69 ± 2.89	59.75 ± 2.95	1–3 wk	2–7 d	8 wk	②④
Xu YH	2016	41/41	64.0 ± 1.7	63.4 ± 1.7	3–4 wk	2–7 d	4 wk	①③
Guo J	2016	40/40	68.1 ± 8.9	57.8 ± 8.8	3–4 wk	2–7 d	6 wk	③
Li Yang	2017	24/12	58.765 ± 9.68	59.04 ± 9.87	Unclear	Unclear	Unclear	③
Tang FJ	2022	43/43	65.28 ± 9.41	66.54 ± 9.29	3–4 wk	48 h–7 d	4 wk	①②③
Ma Z	2013	35/35	67.2 ± 3.8	67.6 ± 3.2	1–3 wk	48 h	6 wk	①②③
Wang J	2020	48/48	68.74 ± 3.02	68.77 ± 3.01	3 d–2 wk	72 h	Unclear	①②④
Liu XL	2019	53/54	70.14 ± 6.30	71.34 ± 5.16	3 wk	72 h	4 wk	①②
Wang L	2014	35/35	Unclear	Unclear	3–4 wk	2–7 d	4 wk	①
Ma FH	2016	70/24	Unclear	Unclear	1–4 wk	48 h–7 d	3 mo	①③
Zhu BH	2012	42/43	Unclear	Unclear	4 wk	72 h	Unclear	④

Outcome indexes: ① NIHSS score; ② FMA score; ③ MBI score; ④ BI score.

BI = Barthel Index, FMA = Fugl-Meyer Assessment, MBI = Modified Barthel Index, NIHSS = National Institutes of Health Stroke Scale.

### 3.3. Risk-of-bias assessment

**Figure 2.**
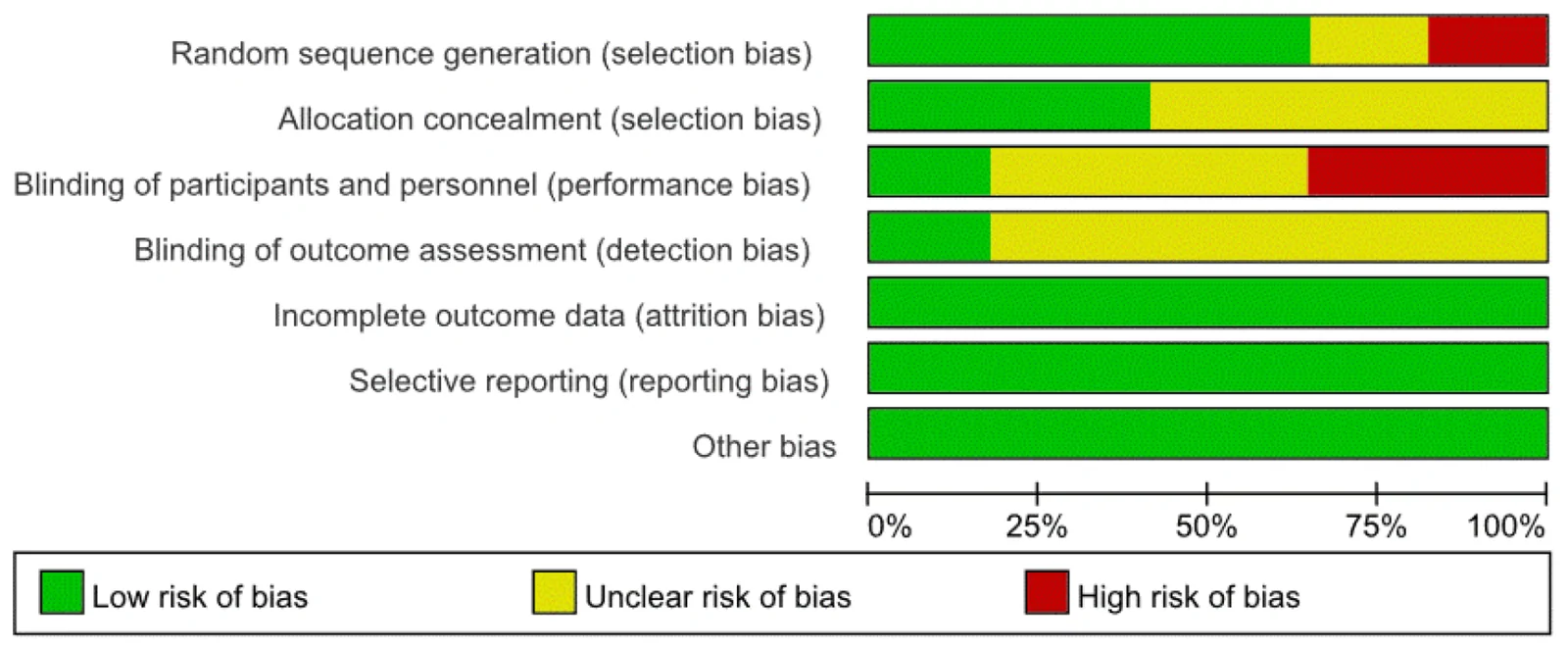
Risk of bias graph.

**Figure 3.**
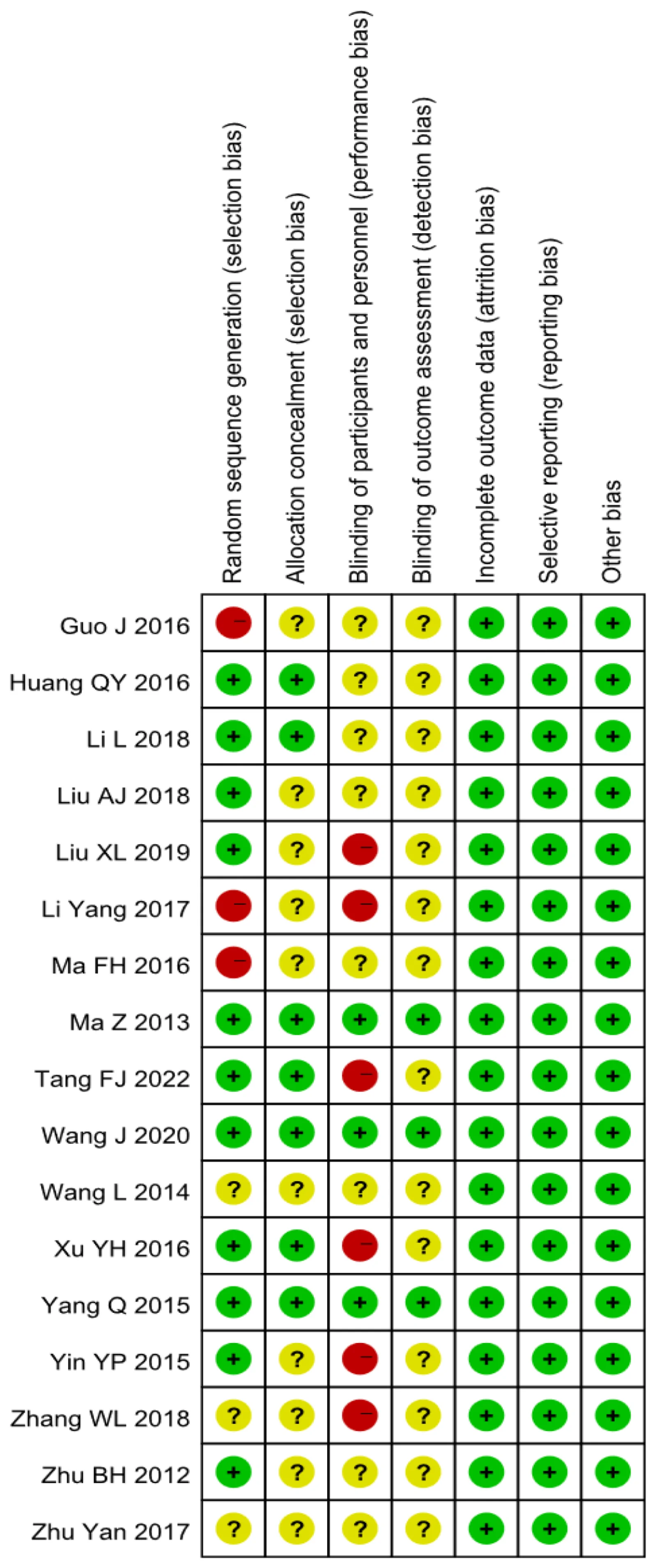
Risk of bias summary.

The methodological quality of the 17 included studies was assessed using the Cochrane risk-of-bias tool. For random sequence generation, 11 studies were judged to be at low risk, 3 at high risk, and 3 at unclear risk. For allocation concealment, 7 studies were judged to be at low risk, whereas the remaining studies did not provide sufficient detail and were rated as unclear risk. With regard to blinding, 3 studies were assessed as low risk, 6 as high risk, and 8 as unclear risk. Outcome completeness was generally acceptable across the included trials, whereas selective reporting and other potential sources of bias were often unclear. The detailed risk-of-bias assessments are presented in Figures 2 and 3.

### 3.4. Meta-analysis of NIHSS

**Figure 4.**
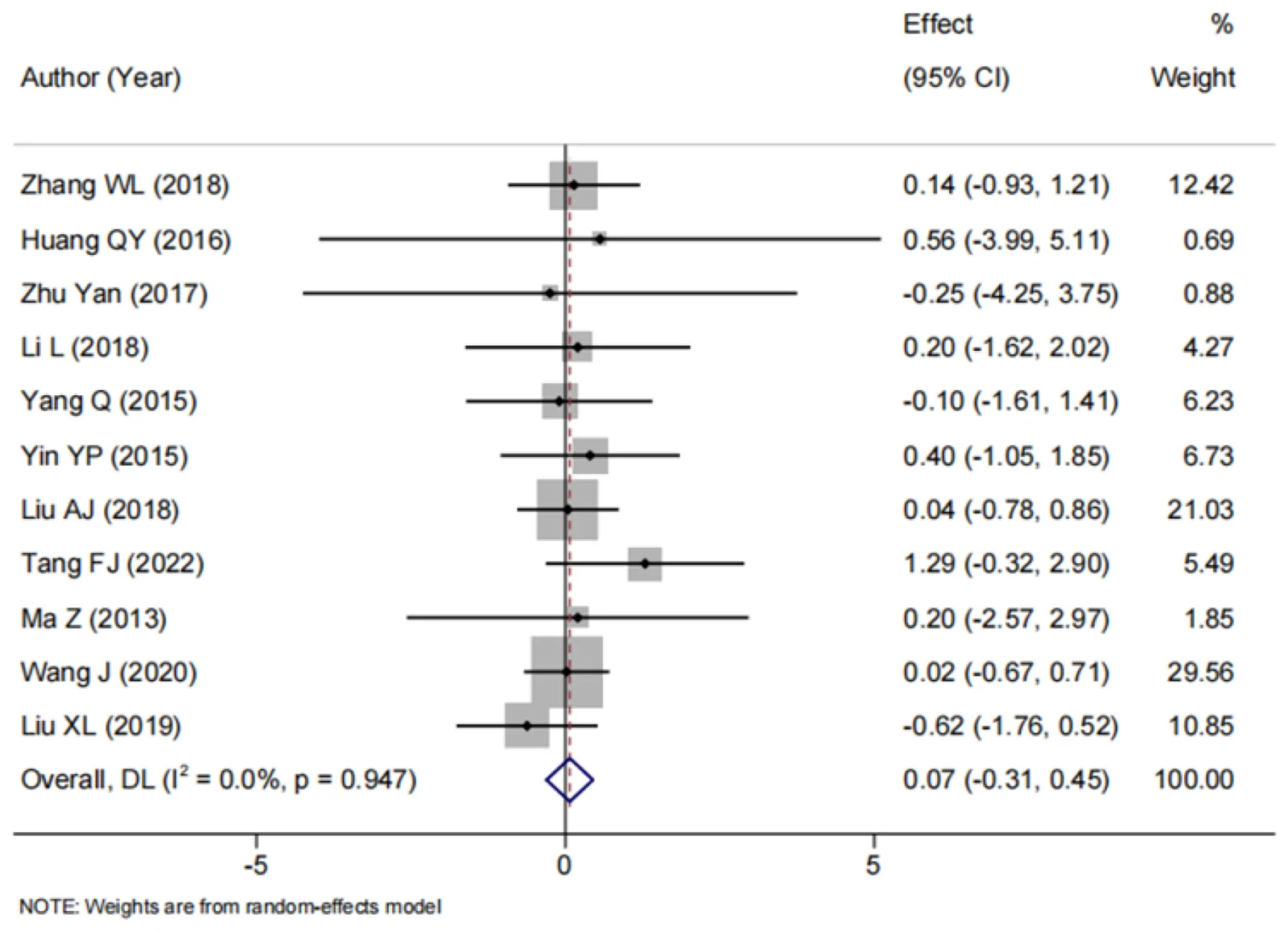
Baseline consistency test of NIHSS score. CI = confidence interval, DL = DerSimonian–Laird, NIHSS = National Institutes of Health Stroke Scale.

Eleven studies evaluated the effect of rehabilitation timing on NIHSS scores after ICH.^[16,17,19,20,23,26–31]^ Before pooling post-intervention outcomes, baseline comparability between the very early and early rehabilitation groups was examined. The baseline NIHSS data showed acceptable homogeneity and no significant between-group difference (pooled effect size = 0.070, *P* = .716), indicating that the 2 groups were comparable at baseline. The baseline consistency analysis is shown in Figure 4.

**Figure 5.**
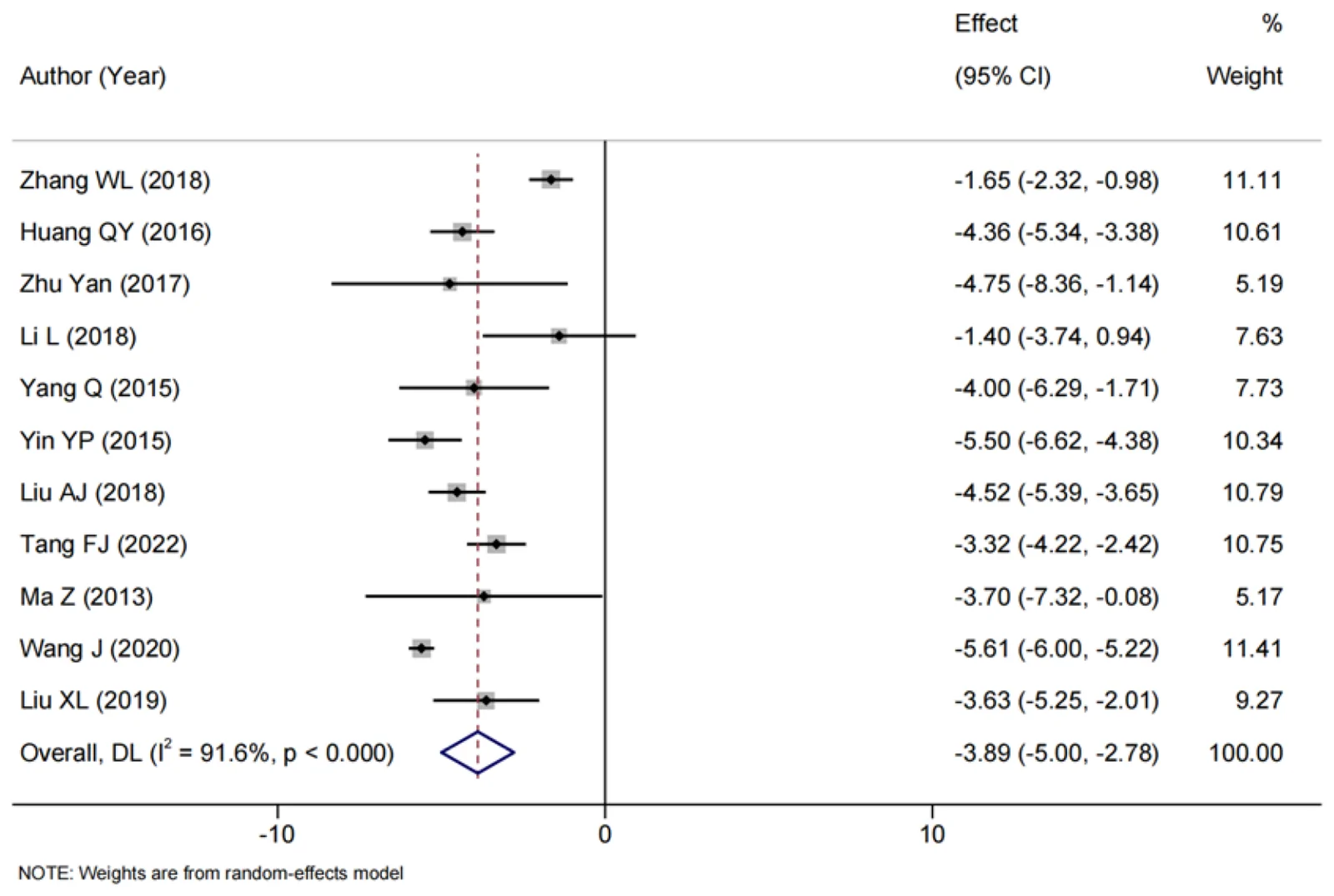
Forest plot of NIHSS score meta-analysis. CI = confidence interval, DL = DerSimonian–Laird, NIHSS = National Institutes of Health Stroke Scale.

Pooled analysis of post-intervention NIHSS scores showed substantial heterogeneity among studies (*I*^2^ = 91.6%, *P* < .001); therefore, a random-effects model was applied. The pooled result showed a significant advantage for the very early rehabilitation group (MD = −3.89, 95% CI: −5.00 to −2.78, *P* < .05), indicating lower NIHSS scores and better neurological recovery in the very early group than in the early group. The corresponding forest plot is shown in Figure 5.

**Figure 6.**
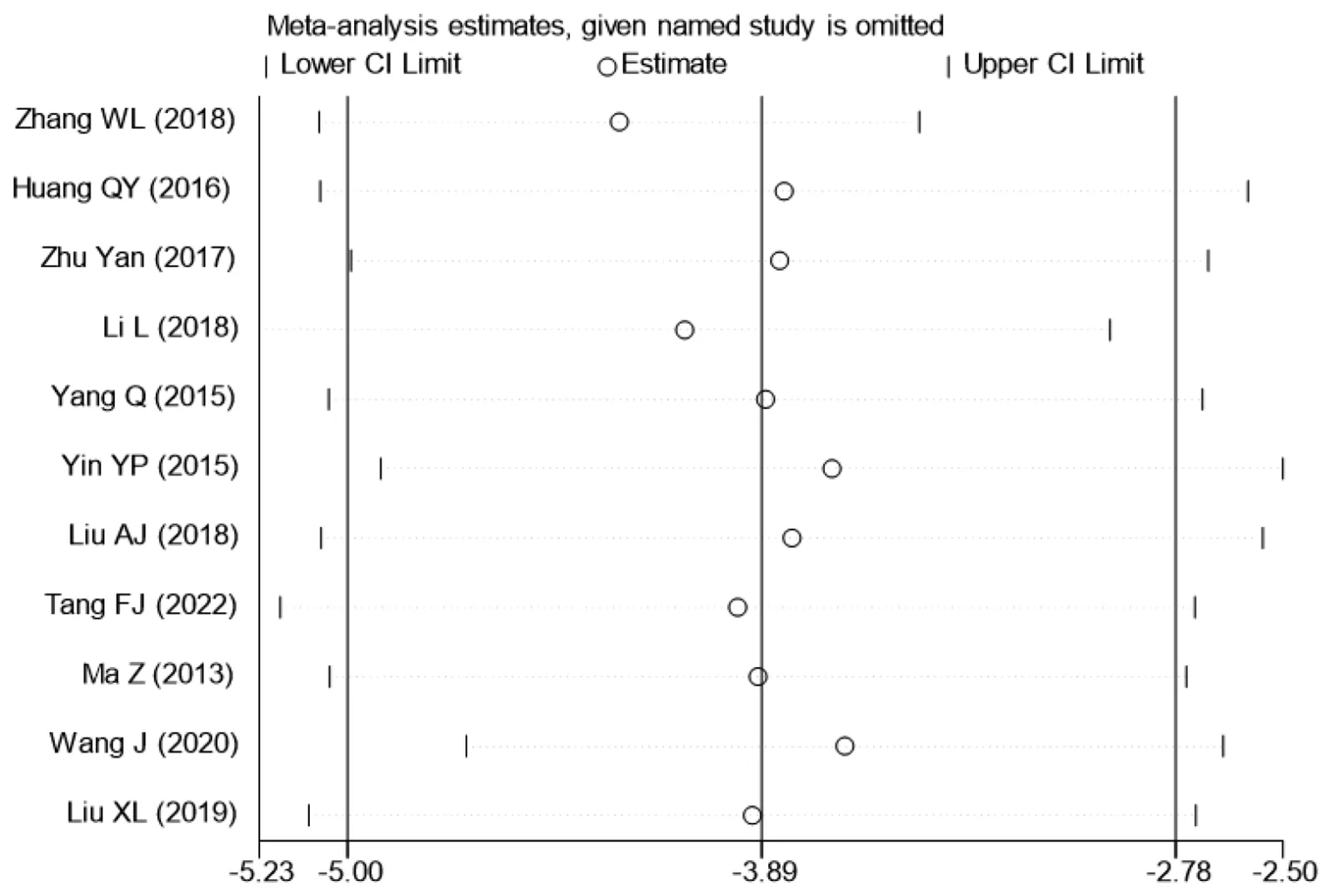
Sensitivity analysis of NIHSS score. CI = confidence interval, NIHSS = National Institutes of Health Stroke Scale.

Sensitivity analysis of the 11 studies showed that sequential exclusion of individual studies did not materially alter the pooled effect estimate, suggesting that the NIHSS result was relatively stable. The sensitivity analysis is shown in Figure 6.

**Figure 7.**
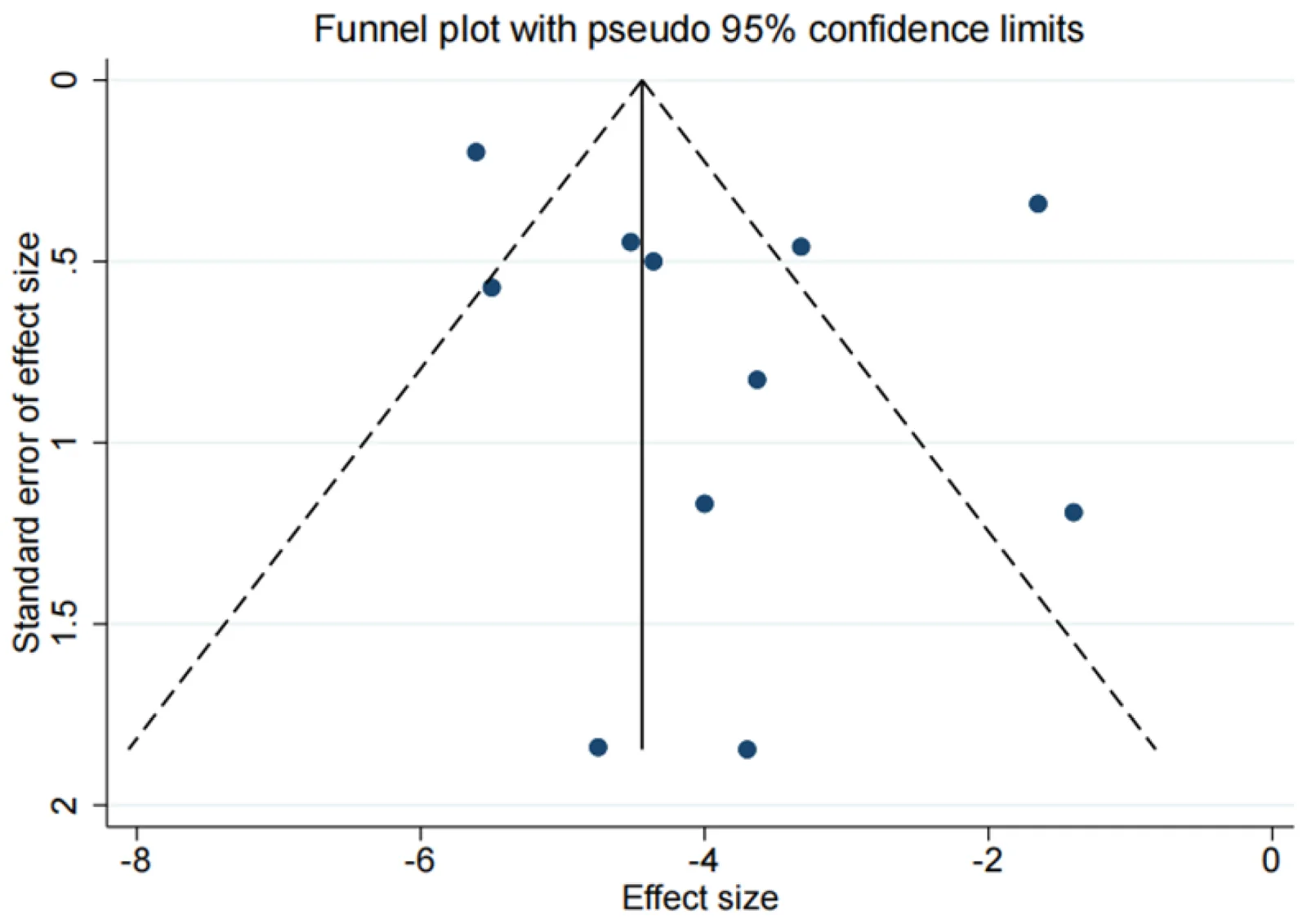
Funnel plot publication bias analysis of NIHSS score. NIHSS = National Institutes of Health Stroke Scale.

Publication bias was assessed for the NIHSS outcome. Visual inspection of the funnel plot suggested approximate symmetry, and further statistical testing showed no significant publication bias (*P* = .306). The funnel plot is shown in Figure 7.

### 3.5. Meta-analysis of FMA

**Figure 8.**
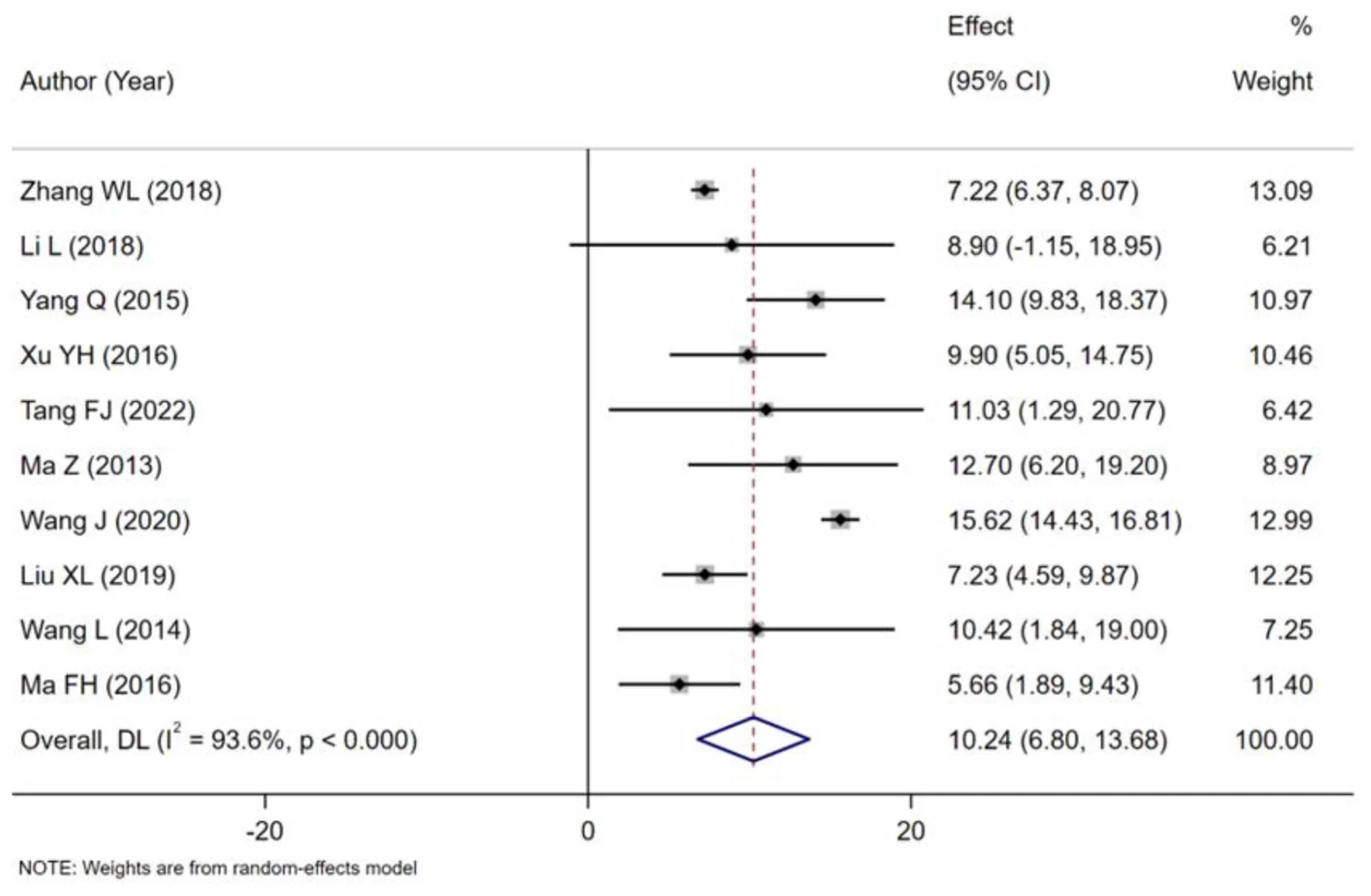
Forest plot of FMA score meta-analysis. CI = confidence interval, DL = DerSimonian–Laird, FMA = Fugl-Meyer Assessment.

Eleven studies reporting FMA outcomes were included to evaluate motor recovery after rehabilitation initiated at different time points.^[16–22,26–29]^ Heterogeneity testing showed substantial between-study heterogeneity (*I*^2^ = 93.6%, *P* < .001); therefore, a random-effects model was applied. The pooled analysis demonstrated that the very early rehabilitation group had significantly higher FMA scores than the early rehabilitation group (MD = 10.24, 95% CI: 6.80–13.68, *P* < .05), indicating better motor recovery with very early rehabilitation. The corresponding forest plot is shown in Figure 8.

### 3.6. Meta-analysis of MBI

**Figure 9.**
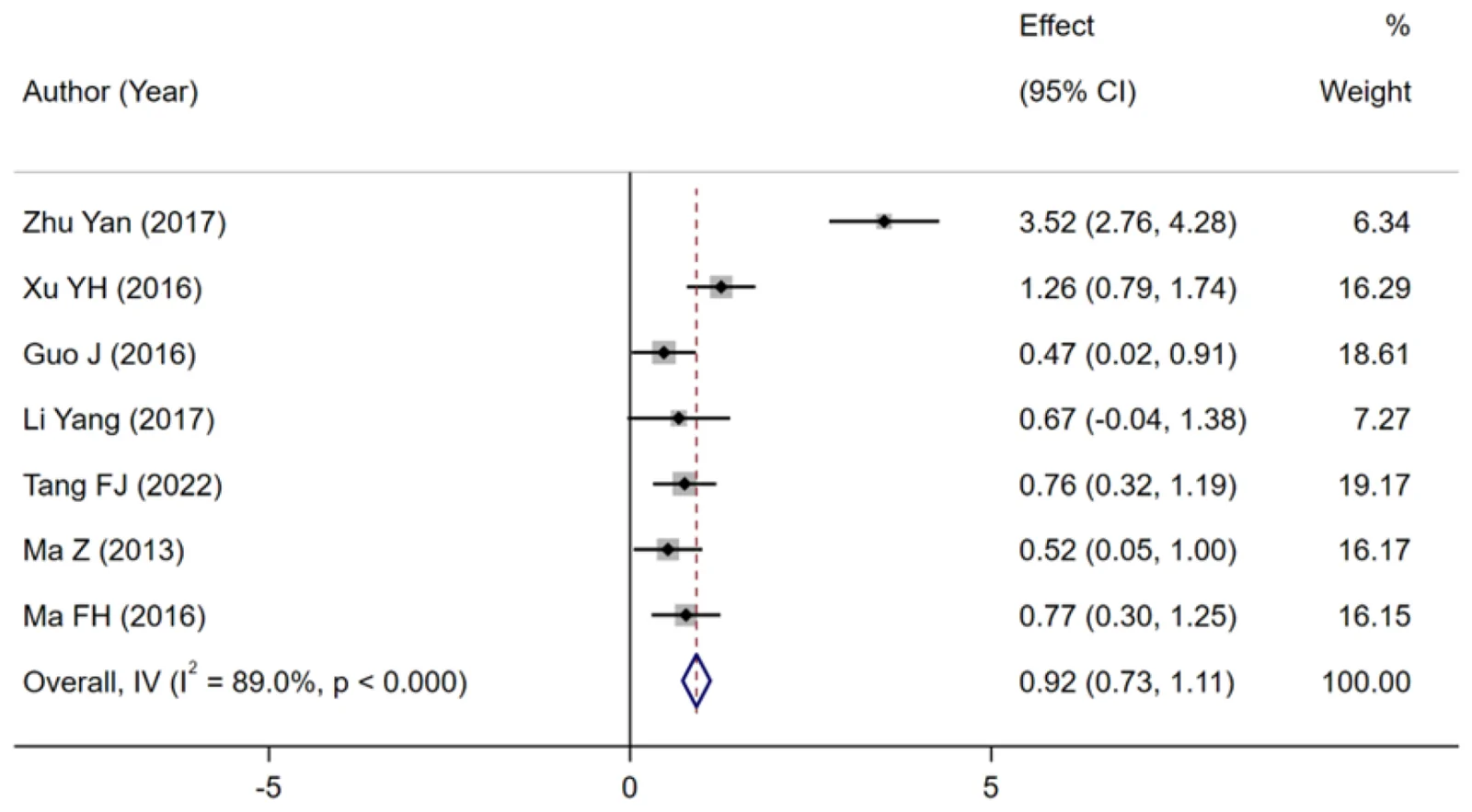
Forest plot of MBI score meta-analysis. CI = confidence interval, MBI = Modified Barthel Index.

Seven studies evaluated the effect of rehabilitation timing on MBI scores.^[18,23–27,31]^ Substantial heterogeneity was observed across the included studies (*I*^2^ = 89.0%, *P* < .001), and a random-effects model was used for the meta-analysis. The pooled result showed that the very early rehabilitation group had significantly higher MBI scores than the early rehabilitation group (MD = 0.92, 95% CI: 0.73–1.11, *P* < .05), indicating better performance in activities of daily living in the very early rehabilitation group. The corresponding forest plot is shown in Figure 9.

### 3.7. Meta-analysis of BI

**Figure 10.**
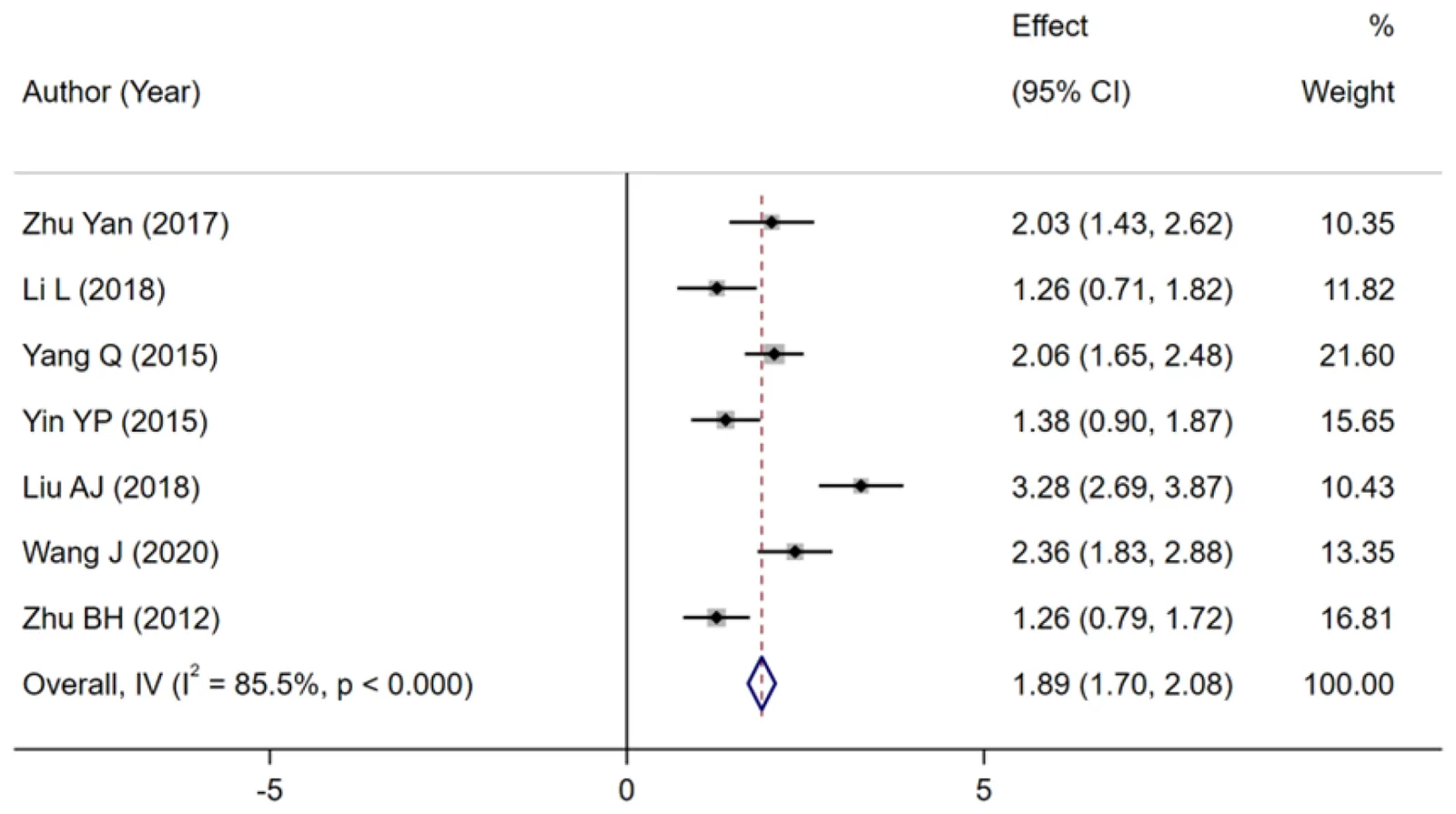
Forest plot of BI score meta-analysis. BI = Barthel Index, CI = confidence interval.

Seven studies reported BI outcomes.^[18–22,28,32]^ Heterogeneity testing indicated substantial between-study heterogeneity (*I*^2^ = 85.5%, *P* < .001), and a random-effects model was therefore applied. The pooled analysis showed that the very early rehabilitation group had significantly higher BI scores than the early rehabilitation group (MD = 1.89, 95% CI: 1.70–2.08, *P* < .05), suggesting better functional independence with very early rehabilitation. The corresponding forest plot is shown in Figure 10.

### 3.8. Safety outcomes

Safety outcomes were not consistently or comprehensively reported across the included trials. Although several studies mentioned clinical stability or monitoring during rehabilitation, standardized data on rebleeding, hematoma expansion, hemodynamic instability, neurological deterioration, falls, or rehabilitation-related adverse events were generally unavailable. Therefore, a quantitative meta-analysis of safety outcomes could not be performed. This lack of standardized safety reporting limits the ability to draw firm conclusions regarding the safety profile of very early rehabilitation after ICH.

### 3.9. Certainty of evidence

Using the GRADE approach, the certainty of evidence was rated as low for NIHSS, FMA, MBI, and BI. The certainty was downgraded mainly because of methodological limitations in the included trials and substantial unexplained heterogeneity across studies. The summary of findings is presented in Table 2.

**Table 2:** GRADE summary of findings.

Outcome	No. participants (studies)	Effect estimate (MD, 95% CI)	Certainty of the evidence (GRADE)	Comments
NIHSS score^‡^	937 (11)	−3.89 (−5.00 to −2.78)	Low^*^,^†^	Very early rehabilitation may improve neurological deficits.
FMA score	949 (11)	10.24 (6.80–13.68)	Low^*^,^†^	Very early rehabilitation may improve motor recovery.
MBI score	520 (7)	0.92 (0.73–1.11)	Low^*^,^†^	Very early rehabilitation may improve activities of daily living.
BI score	639 (7)	1.89 (1.70–2.08)	Low^*^,^†^	Very early rehabilitation may improve functional independence.

Comparison: Very early exercise rehabilitation versus early exercise rehabilitation in patients with intracerebral hemorrhage.

BI = Barthel Index, CI = confidence interval, FMA = Fugl-Meyer Assessment, GRADE = Grading of Recommendations Assessment, Development and Evaluation, MBI = Modified Barthel Index, MD = mean difference, NIHSS = National Institutes of Health Stroke Scale.

* Downgraded 1 level for risk of bias because allocation concealment and blinding were insufficiently reported in several included trials.

† Downgraded 1 level for inconsistency because substantial unexplained heterogeneity was observed across studies.

‡ Publication bias was formally explored only for NIHSS in the manuscript.

## 4. Discussion

ICH is a severe cerebrovascular disease characterized by acute onset, rapid progression, and high mortality and disability, particularly in middle-aged and older adults.^[1–3]^ In routine clinical practice, rehabilitation is often initiated conservatively after stroke onset, but delayed mobilization may miss part of the critical window for neuroplastic recovery and may contribute to slower functional improvement and reduced rehabilitation potential.^[6–8]^ In addition, prolonged bed rest may adversely affect the musculoskeletal, cardiovascular, respiratory, and immune systems, thereby further delaying recovery.^[11,13]^

Very early exercise rehabilitation may offer clinical benefit by taking advantage of the early window of neuroplasticity after brain injury. Early mobilization may promote cortical reorganization, enhance compensatory neural mechanisms, support axonal regeneration, and facilitate the formation of new synaptic connections, all of which may contribute to improved neurological and functional recovery.^[6,8–10]^ However, the initiation of rehabilitation in the hyperacute phase remains controversial. Excessively early mobilization, particularly in patients with unstable hemodynamics or evolving hematoma, may increase the risks of blood pressure fluctuation, cerebral perfusion instability, falls, and possible rebleeding.^[7,13]^ Therefore, rehabilitation timing after ICH should be individualized on the basis of clinical stability rather than determined by time alone.

Although the present meta-analysis primarily compared very early rehabilitation (within 1 week) with early rehabilitation (within 1 month), it is also important to consider the broader clinical context of later rehabilitation. In general, late rehabilitation tends to focus more on compensatory strategies and the prevention of secondary complications than on maximizing the early phase of neurological restitution. By contrast, very early rehabilitation may better capitalize on the peak period of neuroplasticity, whereas early rehabilitation may represent a more conservative approach with potentially lower acute hemodynamic risk. Because late rehabilitation was not directly pooled as a separate comparator in this study, this point should be interpreted as contextual clinical discussion rather than a direct comparative finding of the present meta-analysis.

This meta-analysis included 17 randomized controlled trials involving 1396 patients and compared very early and early exercise rehabilitation after ICH. The pooled results showed that very early rehabilitation was associated with better outcomes in NIHSS, FMA, MBI, and BI scores than early rehabilitation. These findings suggest that, in clinically stable patients, very early mobilization and structured exercise rehabilitation may provide greater benefit for neurological recovery and activities of daily living. However, these findings should be interpreted cautiously because substantial heterogeneity was observed across all major pooled outcomes, and important clinical variables were not consistently reported in the included studies. In particular, the timing categories used in this review were broad operational groupings, and the “very early” category itself included clinically distinct initiation periods such as immediate mobilization, within 48 to 72 hours, and 2 to 7 days.

When designing rehabilitation strategies for middle-aged and older patients with ICH, clinicians should also consider both hemorrhagic and thrombotic risks. Although the major acute concern after ICH is hematoma expansion or rebleeding, patients with advanced age and multiple vascular risk factors may have a substantial thrombotic burden. The congestive heart failure, hypertension, age ≥75 years, diabetes mellitus, stroke/transient ischemic attack/thromboembolism, vascular disease, age 65 to 74 years, and sex category score, originally developed for thromboembolic risk stratification in atrial fibrillation, has also been investigated as a broader marker of cardiovascular and cerebrovascular risk in older populations: a score ≥5 has been associated with increased all-cause mortality and rehospitalization in heart failure patients aged ≥75 years with or without atrial fibrillation, and higher scores have been associated with poorer executive-function performance in community-dwelling older adults.^[33,34]^ Therefore, individualized rehabilitation timing after ICH should consider not only hematoma stability, blood pressure control, and neurological status but also the patient’s global vascular and thrombotic risk profile.

Given the high long-term disability burden associated with ICH, hospital-based very early rehabilitation should be considered only the initial phase of a broader continuum of care. Telemedicine has been described as the remote delivery of clinical services and may provide a framework for extending rehabilitation assessment, monitoring, and coaching beyond hospital-based care.^[35]^ In post-stroke rehabilitation, telerehabilitation, mobile health platforms, smart devices, and mobile applications may help support remote supervision, symptom or risk surveillance, adherence tracking, activity monitoring, and feedback-guided intervention adjustment.^[36–41]^ Evidence from lifestyle intervention sub-studies using mobile technology in high cardiovascular-risk populations suggests that mobile applications and smart devices can improve peak oxygen uptake, heart rate variability, and step-count-related atherosclerotic cardiovascular disease risk indicators.^[42–44]^ Although these digital strategies were not directly assessed in the trials included in this meta-analysis and should not be considered direct evidence for patients with ICH, they support future post-ICH rehabilitation models that integrate supervised exercise, objective activity goals, and physiologic safety monitoring.

## 5. Limitations

Several limitations of this meta-analysis should be acknowledged. First, the mean age of participants in studies with available age data was approximately 64 years, and age data were not reported in several included trials. This may limit the age-specific generalizability of our findings, particularly to very elderly patients. Second, substantial heterogeneity was observed across the primary analyses, with *I*^2^ values ranging from 85.5% to 93.6%. Because the original trials did not consistently report hematoma volume, baseline severity, surgical status, rehabilitation intensity, frequency, progression, supervision methods, or safety-monitoring protocols, we were unable to perform reliable subgroup analyses or meta-regression to distinguish narrower biological phases within the “very early” rehabilitation window (e.g., <24–48 hours vs 3–7 days) or to quantify intervention dose. Third, the included trials did not provide standardized or comprehensive reporting of key safety outcomes, such as rebleeding, hemodynamic instability, neurological deterioration, and falls; therefore, the safety profile of very early rehabilitation could not be quantitatively synthesized. Fourth, the certainty of evidence was rated as low for all primary outcomes, mainly because of risk of bias and substantial inconsistency. Future large-scale, high-quality randomized controlled trials with clearly defined time strata, standardized intervention protocols, objective monitoring, comprehensive safety assessments, and adequate reporting of allocation concealment and blinding are needed to confirm and refine these findings.

A further limitation concerns the search timing. Three randomized trials on early or ultra-early rehabilitation after intracerebral hemorrhage were published after the prespecified search date and were summarized as post-search evidence in Table 3.^[45–47]^ Although all 3 were directionally concordant with the findings of the present meta-analysis, their timing comparators ranged from 12 hours to 7 days and did not map uniformly onto the original “very early versus early” contrast. One trial also used a non-NIHSS neurological deficit scale and reported unusually narrow dispersion values for several functional outcomes. These differences further illustrate the heterogeneity of rehabilitation timing definitions already noted above and preclude valid pooling without a full update of the review process. The present results should therefore be interpreted as reflecting the evidence available up to the prespecified search date, and a fully updated systematic review and meta-analysis is warranted to formally integrate these emerging data.

**Table 3:** Characteristics and findings of randomized controlled trials identified in the post-search evidence check (studies published after the prespecified search date of October 10, 2023).

First author (year)	Design/population	Timing contrast (earlier vs later)	n (earlier/later)	Outcomes reported (earlier vs later)	Direction of effect
Zeng (2026)^[45]^ China Medical Innovation, 23(7):33–36	RCT; ICH after minimally invasive hematoma evacuation	48 vs 72 h post-op	55/55	NIHSS: 10.16 ± 2.30 vs 11.62 ± 2.59 FMA: 71.39 ± 9.11 vs 66.71 ± 8.51 BI: 76.32 ± 12.59 vs 69.78 ± 10.59 (posttreatment, 3 mo; all *P* < .05)	Favors earlier initiation
Wang (2025)^[46]^ Chin J Mod Drug Appl, 19(10):160–163	RCT; hypertensive ICH with hemiplegia (surgical)	48 h vs 7 d	35/35	NIHSS (28 d): 4.2 ± 2.0 vs 6.3 ± 2.8 FMA-UE (12 wk): 44.3 ± 7.5 vs 39.5 ± 8.4 FMA-LE (12 wk): 24.6 ± 4.3 vs 22.0 ± 5.6 (all *P* < .05)	Favors earlier initiation
Kuang (2025)^[47]^ J Shantou Univ Med Coll, 38(3):169–173	RCT; hemorrhagic stroke, SAH excluded (rehabilitation nursing)	12 h after admission vs 48 h after stabilization	128/130	Neuro deficit score* (30 d): 10.33 ± 0.26 vs 14.63 ± 0.22 FMA (30 d): 51.68 ± 0.19 vs 39.85 ± 0.30 BI (30 d): 60.55 ± 0.24 vs 47.72 ± 0.18 QLI (30 d): 7.59 ± 0.11 vs 5.88 ± 0.09 (all *P* < .001)	Favors earlier initiation

Values are presented as mean ± standard deviation for the earlier-initiation group versus the later-initiation group, as reported in the original articles.

These trials were identified after the prespecified search date of October 10, 2023, and were treated as post-search evidence. They were not incorporated into the quantitative synthesis because their timing comparators (48 vs 72 h, 48 h vs 7 d, and 12 vs 48 h) did not map uniformly onto the original “very early versus early” contrast. Formal integration of these studies would require a full update of the systematic review process, including renewed study selection, data extraction, risk-of-bias assessment, GRADE evaluation, and reanalysis of pooled estimates. All 3 trials were directionally consistent with the pooled findings of the present review.

BI = Barthel Index, FMA = Fugl-Meyer Assessment, FMA-UE/FMA-LE = upper-/lower-extremity FMA subscores, ICH = intracerebral hemorrhage, NIHSS = National Institutes of Health Stroke Scale, QLI = quality of life index, RCT = randomized controlled trial, SAH = subarachnoid hemorrhage.

* Kuang (2025) used a Chinese clinical neurological deficit scale based on the 1995 national criteria rather than the NIHSS; therefore, this outcome was not directly comparable with the NIHSS outcome in the main meta-analysis. Several functional outcomes in this trial also showed unusually narrow dispersion values. To maintain consistency with the prespecified outcome framework and avoid disproportionate influence on pooled estimates, these data were summarized descriptively.

## 6. Conclusion

In conclusion, this meta-analysis suggests, with low certainty of evidence, that very early exercise rehabilitation may be associated with greater improvement in neurological function, motor recovery, and activities of daily living than rehabilitation initiated later within the early phase in clinically stable patients with ICH. However, because the available evidence is limited by substantial heterogeneity, variable study quality, and incomplete safety reporting, these findings should be interpreted cautiously. Very early rehabilitation should therefore be considered within the framework of individualized clinical assessment rather than as a universal strategy for all patients. Further high-quality randomized controlled trials are needed to define the optimal initiation window, intervention intensity, and safety boundaries of post-ICH rehabilitation more precisely.

## Author contributions

**Conceptualization:** Taiqiang Kan, Bensi Zhang.

**Data curation:** Taiqiang Kan, Lining Ding, Siqi Wang, Yunfei Li, Bensi Zhang.

**Formal analysis:** Taiqiang Kan, Lining Ding, Siqi Wang, Tong Li, Zhuang Li, Bensi Zhang.

**Funding acquisition:** Bensi Zhang.

**Investigation:** Taiqiang Kan, Lining Ding, Siqi Wang, Yunfei Li, Bensi Zhang.

**Methodology:** Taiqiang Kan, Siqi Wang, Tong Li, Bensi Zhang.

**Project administration:** Bensi Zhang.

**Resources:** Chenbang Ma, Zhuang Li.

**Software:** Taiqiang Kan.

**Supervision:** Bensi Zhang.

**Validation:** Lining Ding, Tong Li, Zhongqin He, Chun Shi.

**Visualization:** Taiqiang Kan.

**Writing – original draft:** Taiqiang Kan.

**Writing – review & editing:** Taiqiang Kan, Zhongqin He, Chun Shi, Chenbang Ma, Bensi Zhang.

## References

[1] Parry-Jones AR Krishnamurthi R Ziai WC. World Stroke Organization (WSO): global intracerebral hemorrhage factsheet 2025. Int J Stroke. 2025;20:145–50. 39629687 10.1177/17474930241307876 PMC11786522

[2] GBD 2021 Stroke Risk Factor Collaborators. Global, regional, and national burden of stroke and its risk factors, 1990–2021: a systematic analysis for the global burden of disease study 2021. Lancet Neurol. 2024;23:973–1003. 39304265 10.1016/S1474-4422(24)00369-7 PMC12254192

[3] Wang Y-J Li Z-X Gu H-Q. China stroke statistics: an update on the 2019 report from the national center for healthcare quality management in Neurological Diseases, China National Clinical Research Center for Neurological Diseases, the Chinese Stroke Association, national center for chronic and non-communicable disease control and prevention, Chinese center for disease control and prevention and institute for global neuroscience and stroke collaborations. Stroke Vasc Neurol. 2022;7:415–50. 35443985 10.1136/svn-2021-001374 PMC9614174

[4] Greenberg SM Ziai WC Cordonnier C. 2022 guideline for the management of patients with spontaneous intracerebral hemorrhage: a guideline from the American Heart Association/American Stroke Association. Stroke. 2022;53:e282–361. 35579034 10.1161/STR.0000000000000407

[5] Lee KE Choi M Jeoung B. Effectiveness of rehabilitation exercise in improving physical function of stroke patients: a systematic review. Int J Environ Res Public Health. 2022;19:12739. 36232038 10.3390/ijerph191912739 PMC9566624

[6] Kwakkel G Stinear C Essers B. Motor rehabilitation after stroke: European Stroke Organisation (ESO) consensus-based definition and guiding framework. Eur Stroke J. 2023;8:880–94. 37548025 10.1177/23969873231191304 PMC10683740

[7] Kakuda W Nakajima M Oki K. Evidence and recommendations for acute stroke rehabilitation from the japan stroke society: abridged secondary publication of the Japanese-language version. Prog Rehabil Med. 2024;9:20240015. 38660472 10.2490/prm.20240015 PMC11038992

[8] Raghavan P. Top-down and bottom-up mechanisms of motor recovery poststroke. Phys Med Rehabil Clin N Am. 2024;35:235–57. 38514216 10.1016/j.pmr.2023.07.006

[9] Li G Tao X Lei B. Effects of exercise on post-stroke cognitive function: a systematic review and meta-analysis of randomized controlled trials. Top Stroke Rehabil. 2024;31:645–66. 38825881 10.1080/10749357.2024.2356393

[10] Zhou Y Ren H Hou X. The effect of exercise on balance function in stroke patients: a systematic review and meta-analysis of randomized controlled trials. J Neurol. 2024;271:4751–68. 38834700 10.1007/s00415-024-12467-1

[11] Cardoso R Parola V Neves H. Physical rehabilitation programs for bedridden patients with prolonged immobility: a scoping review. Int J Environ Res Public Health. 2022;19:6420. 35682005 10.3390/ijerph19116420 PMC9180781

[12] Yen H-C Jeng J-S Chen W-S. Early mobilization of mild-moderate intracerebral hemorrhage patients in a stroke center: a randomized controlled trial. Neurorehabil Neural Repair. 2020;34:72–81. 31858865 10.1177/1545968319893294

[13] Mariana de Aquino Miranda J Mendes Borges V Bazan R José Luvizutto G Sabrysna Morais Shinosaki J. Early mobilization in acute stroke phase: a systematic review. Top Stroke Rehabil. 2023;30:157–68. 34927568 10.1080/10749357.2021.2008595

[14] Zheng P Zhu X Cadilhac DA Luo Y Liu N. Early rehabilitation after acute intracerebral hemorrhage in china-a need for new research directions and more data: a systematic review. Geriatr Nurs. 2025;64:103339. 40413075 10.1016/j.gerinurse.2025.04.012

[15] Higgins JPT Altman DG Gøtzsche PC. The Cochrane Collaboration’s tool for assessing risk of bias in randomised trials. BMJ. 2011;343:d5928. 22008217 10.1136/bmj.d5928 PMC3196245

[16] Zhang LW. Clinical correlation evaluation of intervention time and therapeutic effect of rehabilitation treatment for intracerebral hemorrhage. Mod Diagn Treat. 2018;29:2414–6.

[17] Huang YQ. Clinical correlation study on intervention time and therapeutic effect of rehabilitation treatment for intracerebral hemorrhage. China Med Pharm. 2016;6:198–201.

[18] Zhu Y. Analysis of the relationship between intervention time of rehabilitation treatment and clinical effect in patients with intracerebral hemorrhage. Biped Health. 2017;26:40.

[19] Li L Wu KX. Study on the effect of intervention time of rehabilitation treatment on therapeutic effect of intracerebral hemorrhage. Med Aesthet Beauty. 2018;27:45.

[20] Yang Q. Clinical study on the effect of rehabilitation intervention timing on functional recovery in 70 patients with intracerebral hemorrhage. Chin J Ethnomed Ethnopharm. 2015;24:113–4.

[21] Yin YP. Effect of different rehabilitation intervention times on functional recovery in elderly patients with intracerebral hemorrhage. Chin Community Dr. 2015;31:156.

[22] Liu AJ. Effects of different rehabilitation intervention times on neurological function and activities of daily living in intracerebral hemorrhage. China Contin Med Educ. 2018;10:148–50.

[23] Xu YH Wei XL Zhang XN. Effect of rehabilitation training intervened at different times on motor function recovery of patients with intracerebral hemorrhage. Med Recapitulate. 2016;22:4762–4.

[24] Guo J Ma FL. Effect of different rehabilitation intervention times on neurological function and activities of daily living in patients with intracerebral hemorrhage. Clin Res Pract. 2016;1:163–4.

[25] Li Y Lv SK Zou KW. Effects of rehabilitation at different intervention timings on hand function and activities of daily living in patients with intracerebral hemorrhage. Health Care Guide. 2017;186:204.

[26] Tang FJ Zhang YL Li LN. Effect of time window intervention of exercise training on physiological function recovery of patients with intracerebral hemorrhage. J Qilu Nurs. 2022;28:84–6.

[27] Ma Z Sun YF Kang LL. Effect of different rehabilitation intervention times on functional recovery in elderly patients with intracerebral hemorrhage. Chin J Gerontol. 2013;33:6261–2.

[28] Wang J. Analysis of the impact of different rehabilitation intervention timing on motor and neurological functions in elderly patients with intracerebral hemorrhage. Reflexol Rehabil Med. 2020;1:127–9.

[29] Liu XL Zhang TX. Effect of different rehabilitation intervention timing on motor and neurological functions in elderly patients with intracerebral hemorrhage. Clin Res Pract. 2019;4:59–60.

[30] Wang L Tang LY. Effect of different rehabilitation intervention times on motor function recovery in patients with intracerebral hemorrhage. Health All. 2014;8:13.

[31] Ma FH. Study on the selection of intervention timing of rehabilitation training for patients with hypertensive intracerebral hemorrhage after craniotomy. J Pract Clin Med. 2016;20:152–3.

[32] Zhu BH. Analysis of curative effect of drug therapy and rehabilitation intervention time in acute stage of intracerebral hemorrhage. Seek Med Ask Med. 2012;10:562–3.

[33] Sonaglioni A Lonati C Rigamonti E. CHA2DS2-VASc score stratifies mortality risk in heart failure patients aged 75 years and older with and without atrial fibrillation. Aging Clin Exp Res. 2022;34:1707–20. 35294768 10.1007/s40520-022-02107-x PMC8925288

[34] Duda BM Keith CM Sweet LH. CHA2DS2-VASc stroke risk index and executive functioning in older adults. Arch Clin Neuropsychol. 2020;35:155–64. 31423534 10.1093/arclin/acz031

[35] Hayiroğlu M. Telemedicine: current concepts and future perceptions. Anatol J Cardiol. 2019;22(Suppl 2):21–2. 10.14744/AnatolJCardiol.2019.12525 31670712

[36] Pitliya A Siddiq AB Oli D. Telerehabilitation in post-stroke care: a systematic review and meta-analysis of randomized controlled trials. Top Stroke Rehabil. 2025;32:323–35. 39172060 10.1080/10749357.2024.2392439

[37] Alwadai B Lazem H Almoajil H Hall AJ Mansoubi M Dawes H. Telerehabilitation and its impact following stroke: an umbrella review of systematic reviews. J Clin Med. 2024;14:50. 39797132 10.3390/jcm14010050 PMC11721391

[38] Liang Q Tao Y He J Bo Y Xu L Zhao F. Effects of home-based telemedicine and mhealth interventions on blood pressure in stroke patients: a systematic evaluation and meta-analysis of randomized controlled trials. J Stroke Cerebrovasc Dis. 2024;33:107928. 39187214 10.1016/j.jstrokecerebrovasdis.2024.107928

[39] Rintala A Kossi O Bonnechère B Evers L Printemps E Feys P. Mobile health applications for improving physical function, physical activity, and quality of life in stroke survivors: a systematic review. Disabil Rehabil. 2023;45:4001–15. 36325613 10.1080/09638288.2022.2140844

[40] Hao J Pu Y Chen Z Siu K-C. Effects of virtual reality-based telerehabilitation for stroke patients: a systematic review and meta-analysis of randomized controlled trials. J Stroke Cerebrovasc Dis. 2023;32:106960. 36586244 10.1016/j.jstrokecerebrovasdis.2022.106960

[41] Laver KE Adey-Wakeling Z Crotty M Lannin NA George S Sherrington C. Telerehabilitation services for stroke. Cochrane Database Syst Rev. 2020;1:CD010255. 32002991 10.1002/14651858.CD010255.pub3 PMC6992923

[42] Hayiroğlu M Çinar T Cilli Hayiroğlu S Şaylik F Uzun M Tekkeşin A. The role of smart devices and mobile application on the change in peak VO_2_ in patients with high cardiovascular risk: a sub-study of the LIGHT randomised clinical trial. Acta Cardiol. 2023;78:1000–5. 37318090 10.1080/00015385.2023.2223005

[43] Hayiroğlu M Çinier G Yüksel G. Effect of a mobile application and smart devices on heart rate variability in diabetic patients with high cardiovascular risk: a sub-study of the LIGHT randomized clinical trial. Kardiol Pol. 2021;79:1239–44. 34599495 10.33963/KP.a2021.0112

[44] Hayiroğlu M Çinar T Çinier G. The effect of 1-year mean step count on the change in the atherosclerotic cardiovascular disease risk calculation in patients with high cardiovascular risk: a sub-study of the LIGHT randomized clinical trial. Kardiol Pol. 2021;79:1140–2. 34506630 10.33963/KP.a2021.0108

[45] Zeng FN. Effect of early rehabilitation therapy on neurological and motor function in patients after minimally invasive evacuation of intracerebral hemorrhage. China Med Innov. 2026;23:33–6.

[46] Wang XL Lin GZ Chen MZ. Effect of early rehabilitation training on neurological function and prognosis in patients with hypertensive intracerebral hemorrhage and hemiplegia. Chin J Modern Drug Appl. 2025;19:160–3.

[47] Kuang CE Gao HL Chen DH Guo P. Effect of very early rehabilitation nursing on functional recovery and quality of life in patients with hemorrhagic stroke. J Shantou Univ Med College. 2025;38:169–73.

